# Visualization of precut DSAEK and pre-stripped DMEK donor corneas by intraoperative optical coherence tomography using the RESCAN 700

**DOI:** 10.1186/s12886-016-0308-z

**Published:** 2016-08-04

**Authors:** Akira Kobayashi, Hideaki Yokogawa, Natsuko Mori, Kazuhisa Sugiyama

**Affiliations:** Department of Ophthalmology, Kanazawa University Graduate School of Medical Science, 13-1 Takara-machi, Kanazawa-shi, Ishikawa-ken 920-8641 Japan

**Keywords:** Descemet’s stripping automated endothelial keratoplasty, Descemet’s membrane endothelial keratoplasty, Intraoperative OCT, RESCAN 700

## Abstract

**Background:**

To report the feasibility of intraoperative spectral domain optical coherence tomography (OCT) using the RESCAN 700 for the visualization and evaluation of precut Descemet’s stripping automated endothelial keratoplasty (DSAEK) and prestripped Descemet’s membrane endothelial keratoplasty (DMEK) donor tissue.

**Methods:**

Precut DSAEK (*n* = 11), prestripped DMEK (*n* = 4) preserved in a viewing chamber were examined by intraoperative OCT. Wholly intact donor corneas for penetrating keratoplasty (PK) (*n* = 8) were also examined as controls. The obtained images were analyzed qualitatively for characteristics of each type of donor.

**Results:**

For each type of donor, characteristic images were consistently obtained by intraoperative OCT in both the front and back views through the viewing chamber. In wholly intact donors for PK, appearance of normal corneal curvature and stromal texture with high reflectivity of epithelium and endothelium cell layers were clearly visualized. In precut DSAEK donors, precut lines were characteristically visualized in addition to the intact donor cornea images. In prestripped DMEK donors, identical OCT images to the intact donor cornea were noted when observed from the anterior surface. However, peripheral partial detachments of Descemet’s membrane were characteristically observed in all prestripped DMEK donors when viewed from the back of the viewing chamber.

**Conclusion:**

Rapid visualization and rough evaluation of donor tissues for PK, precut DSAEK and prestripped DMEK donor corneas by intraoperative OCT was consistently possible through the viewing chamber. Therefore, this device may be used as an alternative of AS-OCT when the eyebank does not have their own AS-OCT. Although the peripheral detachment in DMEK donors are quite common and clinically non-problematic in DMEK donor quality and subsequent DMEK surgeries, it may be useful to distinguish between wholly intact PK donors and prestripped DMEK donors, enabling to prevent mix-ups of donors, especially when several different types of keratoplasties are scheduled in a same day in one operating theater.

## Background

Descemet’s stripping automated endothelial keratoplasty (DSAEK) [1–7] or Descemet’s membrane endothelial keratoplasty (DMEK) has been preferably used for the treatment of corneal endothelial dysfunction [8–17]. Recent statistics showed that these techniques are the most common keratoplasty procedures performed in the U.S. [18]. The use of precut DSAEK tissue [19] or prestripped DMEK tissue [20] supplied by the eyebank has greatly shortened the operation time, avoids a large capital investment in an expensive microkeratome machine for DSAEK and avoids tissue loss due to unsuccessful Descemet’s membrane stripping/harvesting in DMEK. Evaluation of these premade endothelial keratoplasty tissues before surgery can be successfully and effectively performed by slit lamp biomicroscopy and specular microscopy. However, it is difficult to precisely evaluate the depth and quality of the lamellar cut or prestripped status by these modalities.

Previously, anterior segment optical coherence tomography (AS-OCT) has proven useful to screen donor corneas for previous refractive surgeries such as laser in situ keratomileusis [21, 22]. Recently, some eyebanks have adopted AS-OCT as their standard procedures for tissue evaluation [23].

Herein, we show the feasibilities of intraoperative spectral domain OCT using the RESCAN 700 (Carl Zeiss Meditec, Germany) [24, 25] for the rapid visualization and rough evaluation of precut DSAEK and prestripped DMEK donor tissue.

## Methods

The study was approved by the Ethical Committee of Kanazawa University Graduate School of Medical Science and followed the tenets of the Declaration of Helsinki. Internationally shipped precut DSAEK (*n* = 11) and prestripped DMEK (*n* = 4) donor corneas from a US eyebank (SightLife™, Seattle, USA) preserved in a viewing chamber were used. For a control, domestic wholly intact donor corneas taken post-mortem for penetrating keratoplasty (PK) (*n* = 8) were used. Consent was received for the use of these corneas for this research by the donors’ next of kin. The donor cornea for DSAEK was dissected with a microkeratome (ALTK Cbm; Moria, France) equipped with a 300 μm head at the US eyebank. The DMEK donor graft was also prepared at the US eyebank using the submerged cornea using the backgrounds away (SCUBA) technique [15]. All donor tissues (PK tissue, precut DSAEK tissue, pre-stripped DMEK tissue) were kept in Optisol GS solution (Bausch & Lomb Surgical, USA) and internationally shipped by airplane at 4° Celsius. After warming the donor tissues, they were subjected to routine slit lamp biomicroscopy and intraoperative SD-OCT without taking them out of the preservation medium and container.

### Intraoperative OCT

The Rescan 700 is a real-time intraoperative spectral domain OCT integrated into the OPMI Lumera 700 microscope (Carl Zeiss Meditec, Germany). The Rescan 700 scan rate was 27,000 axial scans per second. The axial and transverse resolution in tissue are 5 μm and 15 μm, respectively. Each donor tissue was scanned through the transparent window of the corneal viewing chamber, which was placed on a flat table. The OCT images are recorded in a horizontal and vertical orientation. The scan depth was 2 mm and the scan length was adjusted to each donor cornea tissue between 9 and 11 mm (adjustable between 3–16 mm). The surgeon observed the live, uninterrupted intraoperative OCT images during surgery through a small window adjacent to the operating field. Acquired images were evaluated qualitatively for the shape and reflectivity of the donor cornea tissue.

## Results

For each type of donor (PK, DSAEK, DMEK donor), characteristic images were consistently and easily obtained by intraoperative OCT in both the front and back views through the viewing chamber.

**Fig. 1:**
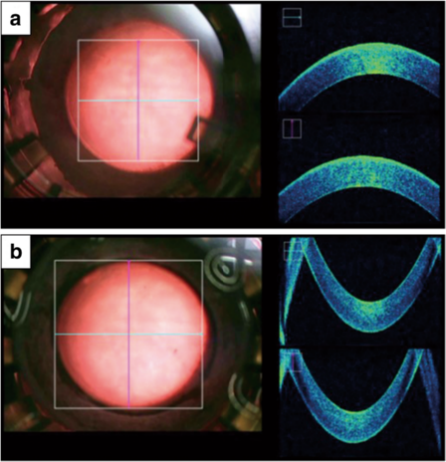
Intraoperative spectral domain optical coherence tomography (SD-OCT) image of a donor cornea for penetrating keratoplasty observed through a viewing chamber. **a** Representative microscope image (surgeon’s view) observed from the anterior (*left*). Normal corneal curvature and stromal texture with high reflectivity of epithelium and endothelium cell layers were observed by intraoperative OCT (*right*). **b** Microscopic image observed from the posterior cornea (*left*) and intraoperative OCT image (*right*)

In wholly intact PK donors, appearance of normal corneal curvature, although not quantitatively measured, and normal stromal texture with high reflectivity of the epithelium and endothelium cell layers were clearly visualized in all donors (n = 8) (Fig. 1).

**Fig. 2:**
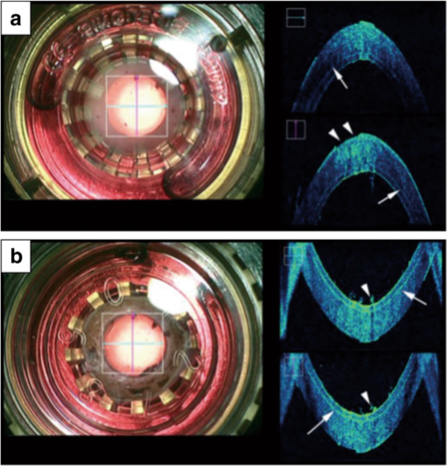
Intraoperative SD-OCT image of a donor cornea for precut Descemet’s stripping automated endothelial keratoplasty (DSAEK) observed through a viewing chamber. **a** Representative microscope image (surgeon’s view) observed from the anterior (*left*). Normal corneal curvature and stromal texture together with high reflectivity of the epithelium, microkeratome cut line (*arrows*), endothelium cell layer and epithelial defect (arrowheads) were observed by intraoperative OCT (*right*). **b** Microscopic image observed from the posterior cornea (*left*) and intraoperative OCT image (*right*). Characteristic microkeratome cut line (*arrows*) together with debris (*arrowheads*) observed on the endothelium cell surface was observed (*left*)

In precut DSAEK donors, precut lines were characteristically visualized rapidly in addition to the PK donor cornea images (Fig. 2). Some precut DSEAK donors showed epithelial defect and/or debris on the surface of endothelium; however, these changes usually do not have any clinical significance.

**Fig. 3:**
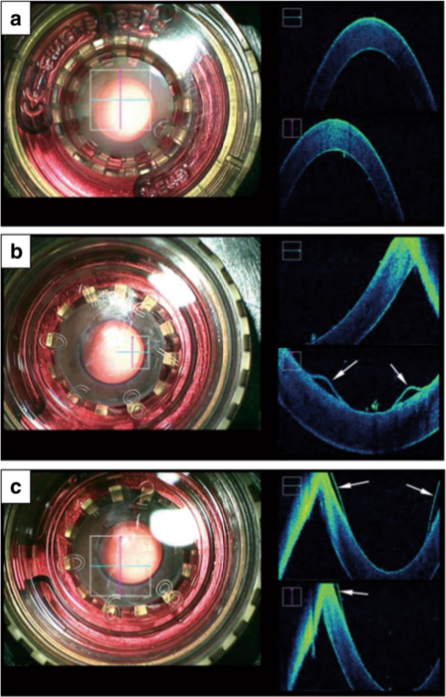
Intraoperative SD-OCT image of a donor cornea for prestripped Descemet’s membrane endothelial keratoplasty (DMEK) observed through a viewing chamber. **a** Representative microscope image (surgeon’s view) observed from the anterior (*left*). Normal corneal curvature and stromal texture were observed by intraoperative OCT (*right*). It was not possible to observe the prestripping line. **b** Microscopic image observed from the posterior cornea (*left*) and intraoperative OCT image (*right*). Characteristic peripheral partial detachment of Descemet’s membrane was observed (*arrows*). **c** Microscopic image of a different area observed from the posterior cornea (*left*) and intraoperative OCT image (*right*). Characteristic peripheral partial detachment of Descemetcpes membrane was also noted (*arrows*)

In prestripped DMEK donors, no characteristic images were noted by SD-OCT when observed from the anterior surface, since peripheral image could not be obtained from the anterior surface observation. However, peripheral partial detachments of Descemet’s membrane were characteristically observed in all prestripped DMEK donors when viewed only from the back of the viewing chamber (Fig. 3). Debris on the surface of endothelium was also seen in some donors.

All donor corneas were transplanted (8 PK, 11 DSAEK, 4 DMEK) to patients, and all corneas remain transparent for at least 12 months.

## Discussion

The microscope integrated intraoperative OCT is a relatively new technology. It enables in vivo 3-D visualization of the steps of retina [26–28] and cornea [29–34] surgeries. The RESCAN 700 is the first commercially available intraoperative OCT system combining a surgical microscope (OPMI LUMERA 700, Carl Zeiss Meditec, Germany) and a spectral domain OCT having a wavelength of 840 nm and a scanning speed of 27,000 A-scans per second. The surgeon does not need to look up from the microscope to see the OCT data since the RESCAN 700 allows the surgeon to simultaneously see the surgical field in both a planar view using the surgical microscope and a cross-sectional view by OCT. Most importantly, images are viewed in real time. The OCT system can be controlled from the microscope’s foot pedal so the surgeon can take videos, snapshots and 3-D OCT images without looking up or stopping the surgery. The usefulness of the RESCAN 700 has been reported for anterior segment surgeries (corneal incisions, scleral closure phacoemulsification groove depth and intraocular lens position) [24], riboflavin penetration in collagen crosslinking [25] and retinal surgeries (evaluation of hyaloid release with triamcinolone and completeness of peel in macular hole and epiretinal membrane) [24]. Recently, we have also confirmed its clinical usefulness in DSAEK for detecting residual fluid between the donor and host cornea which enables a surgeon to decide the appropriate timing to stop corneal massage and push out the residual interface fluid. Also, it is quite useful in DMEK for preventing upside-down DMEK donors in the anterior chamber (data not shown).

Herein, we report the feasibilities of the visualization of donor corneas for PK, precut DSAEK and pre-stripped DMEK by intraoperative OCT using the RESCAN 700. As a result, we showed characteristic images for each type of donor. In wholly intact PK donors, appearance of normal corneal curvature and stromal texture with high reflectivity of the epithelium and endothelium cell layers were clearly visualized. However, the precise corneal curvature could not be measured by RESCAN 700. The images were consistent with those obtained by conventional AS-OCT. In precut DSAEK donors, precut lines were characteristically visualized and the results were also in accordance with those observed by conventional AS-OCT. In prestripped DMEK donors, identical OCT images as the PK donor cornea were noted when observed from the anterior surface. However, peripheral partial detachments of Descemet’s membrane were characteristically observed in all prestripped DMEK donors when viewed from the back of the viewing chamber. Although the peripheral detachment in DMEK donors are quite common and clinically non-problematic in DMEK donor quality and subsequent DMEK surgeries, it may be useful to distinguish between wholly intact PK donors and prestripped DMEK donors by detecting the peripheral detachment sign; it may be difficult or impossible to check the subtle Descemt’s membrane change using slit lamp examination alone.

AS-OCT has been used as a routine procedure for tissue evaluation in some advanced eyebanks to detect previous refractive surgeries and corneal scarring [23]. On the other hand, most eyebanks do not have their own AS-OCT system yet in Japan. Therefore, intraoperative OCT may be used as an alternative device of AS-OCT; it may be able to detect not only corneal donor status, but also corneal scars and previous refractive surgeries just like AS-OCT equipped in eyebank. Another potential usefulness of this device is to routinely check the donor status just before surgery by surgeon to prevent mix-ups of donors, especially when several different types of keratoplasties are scheduled in a same day in one operating theater.

One drawback of this study is that quantitative analysis of the donor status is lacking; the quantitative information such as donor thickness or curvature will definitely strengthen this paper. However, RESCAN 700 currently does not have any quantitative capacity. Development of new software in RESCAN 700 that enables quantitative analysis will enhance the capacity of the device.

## Conclusion

In conclusion, rapid visualization and rough evaluation of donor tissues for PK, precut DSAEK and prestripped DMEK donor corneas by intraoperative OCT was consistently possible through the viewing chamber. Although clinically acceptable, peripheral detachment of Descemet’s membrane of prestripped DMEK donors was successfully and characteristically observed by intraoperative OCT. It may be possible to prevent mix-ups of donors by checking those donors just before surgery by surgeon especially several different types of keratoplasties are scheduled in a same day in one operating theater.

## Abbreviations

OCT: Optical coherence tomography
DSAEK: Descemet’s stripping automated endothelial keratoplasty
DMEK: Descemet’s membrane endothelial keratoplasty
PK: Penetrating keratoplasty
